# Enabling point of care optical diagnostics and treatment of oral lesions in resource-limited settings: preclinical development and evaluation of a low-cost theranostic intraoral device for image-guided photodynamic therapy

**DOI:** 10.1117/1.BIOS.2.4.042305

**Published:** 2025-07-06

**Authors:** Shakir Khan, Bofan Song, Mohammad Ahsan Saad, Danshyl Boodhoo, Sergio Farias, Jonathan M. Trzcinski, Shaobai Li, Lothar Lilge, Brian W. Pogue, Rongguang Liang, Tayyaba Hasan, Jonathan P. Celli

**Affiliations:** a University of Massachusetts Boston, Boston, Massachusetts, United States; b Massachusetts General Hospital, Harvard Medical School, Boston, Massachusetts, United States; c University of Arizona, Wyant College of Optical Sciences, Tucson, Arizona, United States; d University of Toronto, Medical Biophysics, Toronto, Ontario, Canada; e University Health Network, Toronto, Ontario, Canada; f University of Wisconsin-Madison, Madison, Wisconsin, United States; g Harvard University and Massachusetts Institute of Technology, Division of Health Sciences and Technology, Cambridge, Massachusetts, United States

**Keywords:** oral cancer and oral potentially malignant lesions, low- to middle-income countries, smartphone, intraoral imaging, photodynamic therapy, 5-aminolevulinic acid

## Abstract

**Significance:**

Oral squamous cell carcinoma (OSCC) is exceedingly prevalent and deadly in South Asia, especially the Indian subcontinent, affecting 15 out of every 100,000 people and claiming over 70,000 lives annually. This problem is compounded by inadequate medical infrastructure for screening and cancer care, especially in rural areas.

**Aim:**

We sought to build and evaluate a theranostic, low-cost, intraoral device to enable simultaneous multichannel optical imaging and photodynamic therapy (PDT) for the detection and treatment of early oral cancer.

**Approach:**

The system’s performance was assessed using buccal mucosal tissue phantoms containing protoporphyrin IX (PpIX), as well as OSCC *in vitro* tumor models photosensitized with 5-aminolevulinic acid (ALA)-induced PpIX. A murine xenograft model consisting of athymic nude mice implanted with TR146 OSCC cells was used to evaluate the capability of the device to function as a PDT light source with integrated monitoring of PpIX photobleaching as a dosimetry reporter. Experiments were complemented by Monte Carlo modeling of light delivery.

**Results:**

In tissue phantoms the intraoral device reported a linear correlation up to 50  μM PpIX concentration and the extent of photobleaching increased with increasing light dose up to $100\;J/{\mathrm{cm}}^{2}$. PpIX fluorescence contrast was found to improve by using ratiometric analysis of PpIX (red) and autofluorescence (green) fluorescence signals. In mouse models that received PDT treatment via the intraoral device, tumor volume decreased significantly, with histological analysis showing necrosis extending to a depth of 3.0 to 3.5 mm after PDT and consistent with Monte Carlo modeling of light delivery.

**Conclusion:**

A compact, handheld, and low-cost intraoral device with a dental camera form factor is able to carry out simultaneous multichannel white light and fluorescence imaging and image-guided PDT light delivery. The ability to quantify the extent of photobleaching as a dosimetry surrogate points to the potential for real-time treatment monitoring with the same hardware.

Statement of Translation The astronomical incidence of oral cancers in South Asia has been described as a public health crisis. This study introduces optical technology that integrates diagnostic and image-guided photodynamic therapy (PDT) treatment capabilities into a compact, low-cost device engineered for use in global health settings. In addition to the technology itself, this study also reports comprehensive preclinical testing, which contains insights into its use of image guidance and feedback for PDT treatment planning and monitoring with broader applicability to other systems.

## 1 Introduction

Head and neck squamous cell carcinoma (HNSCC) is the sixth most common cancer globally, with ∼900,000 new cases and more than 400,000 mortality.^1^^,^^2^ Among them, two-thirds of oral cancer incidence occurs in low- to middle-income countries (LMICs), especially in the Indian subcontinent (e.g., India, Pakistan, Bangladesh, Sri Lanka, and Nepal),^3^^,^^4^ where oral cancer incidence is most common among men compared with women (third most common). In India, oral cancer comprises 40% of all cancers, driven primarily by the widespread popularity of chewing tobacco-based mixtures (e.g., gutka).^5^ The lack of routine cancer screening contributes to the magnitude of the problem, with 70% of cases diagnosed at late stages (stages III to IV),^6^ making it challenging to provide timely intervention. Even when suspicious lesions are identified, the lack of access to surgical oncology contributes to a mortality rate of 42.4%^7^ and a survival rate of less than 50%.^8^ In addition to tobacco-driven disease, there has also been a sharp increase in cases of human papillomavirus (HPV)-mediated oropharyngeal squamous cell carcinoma (OPSCC) in the United States and Europe.^9^^,^^10^ Even in these high-income regions, unequal access to diagnosis and treatment drives disparities in OPSCC outcomes between patients of differing socioeconomic status as well as certain ethnic and racial groups.^11^^–^^13^ All of these factors point to the need for new technology that enables timely diagnosis and point-of-care treatment of oral lesions, which can be accessible to medically underserved populations worldwide.

Photodynamic therapy (PDT) is a light-based treatment modality that has been shown to be well-suited for the treatment of oral lesions.^14^^,^^15^ In ALA-PDT, 5-aminolevulinic acid (ALA) serves as a photosensitizing precursor, which can be delivered either systemically (intravenously or via oral solution) or topically, leading to accumulation of the photoactive heme precursor protoporphyrin IX (PpIX) somewhat selectively in dysplastic and neoplastic tissues.^15^^–^^18^ Activation of the photosensitizer by delivery of light with appropriate wavelength results in the generation of reactive molecular species that can induce tumor necrosis and disruption of tumor vasculature but with minimal damage to healthy tissue and excellent healing without fibrosis or scarring.^19^ PDT has also been shown to be effective in HPV (−) OPSCC, which is associated with radioresistance,^20^^,^^21^ and also can achieve significant viral load reduction in HPV (+) OPSCC.^22^

In a recent clinical study, we implemented a low-cost, LED-based intraoral PDT light delivery system used in conjunction with systemic ALA photosensitization (via oral solution) for the treatment of early-stage ($T_{1}M_{0}N_{0}$) malignant oral lesions.^18^^,^^23^ This approach was found to be safe, feasible, and clinically effective. Along with the PDT light delivery system, an independent smartphone imaging module consisting of 405 nm excitation LEDs with an emission filter over the phone camera was used to measure the PpIX fluorescence before and after the PDT treatment. This fluorescent imaging module accurately demarcated the lesion margins and provided an estimate of the extent of photobleaching post-PDT. Here, these capabilities are integrated into a single handheld device with a dental camera form factor that provides PDT light delivery and multimodal imaging for diagnostics and monitoring. The hardware retains capabilities of polarized white-light (pWL) and autofluorescence (AF) imaging used in previous clinically validated hardware for oral cancer screening in resource-limited settings.^24^ In a recent clinical study,^25^ this screening device differentiated the carcinoma *in situ* (CIS), dysplasia, and nondysplasia with a sensitivity of 94% [confidence interval (CI): 86.76 to 97.65] and specificity of 72% (CI: 29.04 to 96.33) of cloud-based CNN-AI enabled the imaging device for oral cancer screening in low-resource settings.^26^^,^^27^ Due to its multichannel fluorescence imaging properties, we adapted the screening device for intraoral PDT treatment guidance. Ratiometric fluorescence analysis of PpIX FL at the lesion site relative to lesion autofluorescence showed improved specificity and enhanced margin visualization validated by ultrasound (US) and autofluorescence imaging during PDT.^28^ In this preclinical assessment, the integration of intraoral imaging and image-guided PDT treatment into a single handheld device will pave the way for a complementary streamlined approach for screening and treatment of oral cancer at the point of care.

## 2 Material and Methods

### 2.1 Integrated Intraoral Imaging and PDT Light Delivery Device 

This integrated device builds upon a previous oral diagnostic imaging device that used autofluorescence (AF) and polarized white light (pWL) combined with machine learning, which was clinically validated for classification of suspicious lesions in a screening study of ∼5000 patients in an LMIC setting.^24^^–^^27^ The same handheld imaging hardware was also evaluated in the context of PDT treatment monitoring in patients with early-stage oral cancer.^28^

**Fig. 1:**
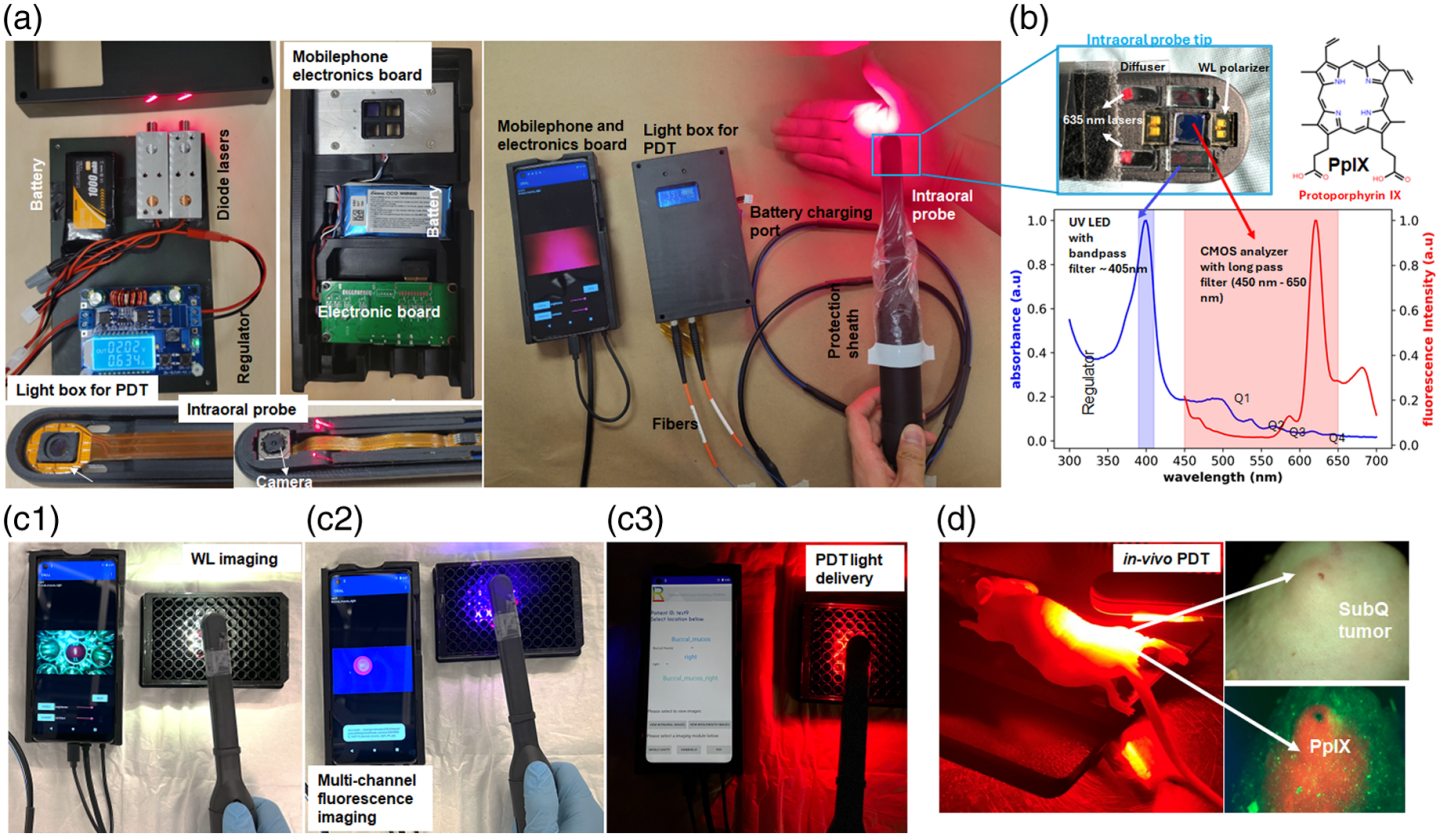
(a) Intraoral device comprises a mobile phone, an electronics board, a lightbox, and an intraoral probe. The lightbox houses a rechargeable battery, two diode lasers, and a current regulator. This regulator provides data on the driving current and voltage. The electronic board and battery control autofluorescence and polarized white light imaging. (b) A closer look at the intraoral probe provides the components with the features of the autofocus camera module, which can operate in autofluorescence and polarized white light imaging modes. Illumination sources include a white light LED, a 405 nm LED for fluorescence excitation, and an integrated laser diode for PDT treatment (c1 to c3). The device’s imaging and image-guided PDT treatment performance is tested in tissue phantoms, cell cultures, and murine xenografts (d).

Here, we report on a handheld intraoral probe that integrates PDT light delivery while retaining the same diagnostic imaging functionality and dental camera form factor as the earlier generation hardware. In brief, it is an intraoral device comprising a mobile phone, an electronics board, a lightbox, and an intraoral probe. As depicted in Fig. 1(a), the lightbox houses a rechargeable battery, two diode lasers, and a current regulator. This regulator provides data on the driving current and voltage. Two red (630 nm) diode lasers are attached to the probe head by multimode fibers. The two mirrors at the tip of the probe head direct the light from the fibers to the tissue surfaces at 90 deg. A light diffusor in front of the probe was applied during PDT to achieve an irradiance of $70\;\mathrm{mW}/{\mathrm{cm}}^{2}$ over a 1 cm diameter beam spot centered on the target tissue and a total fluence of $100\;J/{\mathrm{cm}}^{2}$ at the tissue surface.

Figure 1 and Table 1 show the optical components for white light and fluorescence imaging modes, which use a pWL LED and blue-violet LED, respectively. The micro-camera on the probe’s tip has a low-cost, high-performance 5-megapixel OV5648 CMOS sensor with 2592×1944  pixels (OmniVision Technologies Corp, Santa Clara, California, United States) to achieve reasonable resolution over the large field of view (FOV) in the oral cavity. The phone case attached to the probe has the same optical components, but its camera, positioned on the back of the case, allows for a wide field zoomed-out view of the entire oral cavity using the Moto G5 Android device. Table 1 outlines the system’s overall parameters. For tissue auto-fluorescence imaging,^29^^,^^30^ short-pass filters (425 nm, Asahi Spectra, Tokyo, Japan) and a long-pass filter (450 nm, Asahi Spectra) are positioned in front of the blue-violet LED and camera. Polarized white light (pWL) imaging is used to capture detailed surface information. To eliminate specular reflection, orthogonal linear polarizers (Edmund Optics, Barrington, New Jersey, United States) are mounted in front of each white light LED and in front of the camera module. Four 405 nm Luxeon UV U1 LEDs (from Lumileds, Amsterdam, Netherlands) were added to the back of the smartphone case and the intraoral probe for FL imaging. In addition, four 4000-K Luxeon Z ES LEDs were added for pWL imaging. The 405 nm light aligns with the PpIX Soret band, allowing simultaneous excitation and auto-fluorescence at the lesion site (see Fig. 1). The illumination uniformity of pWL and UV light was determined to be 83.80% and 89.84%, respectively.^25^ The exposure time for pWL and blue-violet light is set to 30 ms to mitigate the photobleaching of PpIX. A working distance of ∼25  mm from the probe to the tissue surface produces the best focus with the probe optics and a field of view of ∼20×30  mm. The device camera generates 1344×1792  pixel dimensions and an 8-bit depth RGB image.

**Table 1:** Intra-oral system performance and characteristics matrices for PpIX, polarized white light imaging (pWLI), autofluorescence imaging (AFI), and PDT.

Parameters by application	AFI and PpIX	pWLI	PDT
Light source	Blue/violet LED	White LED	Diode laser
Detector	CMOS sensor	CMOS sensor	N/A
Spectral output (nm)	405	450 to 650	630 to 635
Detection spectrum (nm)	450 to 650	450 to 650	N/A
Working distance (mm)	10 to 40	10 to 40	N/A
Field of view (at 25mm working distance) (mm)	30 × 20	30 × 20	30 × 20
Lateral resolution (um)	25	25	N/A
Irradiance at lesion surface (${\mathrm{mW}/\mathrm{cm}}^{2}$)	Tunable	Tunable	>30

### 2.2 *In Vivo* and *In Vitro* Models

TR146 cells (Cat. no. ECACC 10032305, source histology: buccal mucosa well-differentiated keratinizing squamous cell carcinoma) were used for both *in vitro* and *in vivo* models to assess the performance of the intraoral device. TR146 was derived from a neck node metastasis of human buccal carcinoma and has been used primarily for *in vitro* cell culture models that resembled normal human buccal epithelium.^31^^,^^32^ Here, TR146 cells were grown in $75\;{\mathrm{cm}}^{2}$ T-flasks (BioLite™ Cell Culture Treated Flasks, vented, Thermo Fisher, #130190, Waltham, Massachusetts, United States) at 37°C, 5% ${\mathrm{CO}}_{2}$, and 95% humidity in Ham’s F-12K (Kaighn’s) medium containing l-glutamine (Corning^®^ #10-080-CV, Corning, New York, United States) and supplemented with 10% FBS, 100  μg/mL penicillin/streptomycin, and 0.5  μg/mL amphotericin-B. The cells in the T75 flask were initially seeded at a concentration of $1\times 10^{4}\;\text{cells}/\mathrm{mL}$, and the F-12K media was changed every 3 to 4 days until cell density was achieved around $0.5\times 10^{6}\;\text{cells}/\mathrm{mL}$.

The cells were harvested by trypsinization (Trypsin, 0.25%) and then resuspended in DPBS (${\mathrm{Ca}}^{2+}$, ${\mathrm{Mg}}^{2+}$ free, Dulbecco’s Phosphate Buffered Saline, HyClone Cytiva, #SH30028.03) for counting. The cells were photosensitized with 3 mM ALA (5-aminolevulinic acid hydrochloride, Sigma Aldrich, #A7793, St. Louis, Missouri, United States) in a new T75 flask after incubation for 4 h to obtain optimum PpIX production.^23^^,^^28^ After harvesting the cells in PBS, $6-10\times 10^{6}$ cells were formed into a pellet after 5 min centrifugation at 500×g for insertion into tissue phantoms. The device’s fluorescence image-based PpIX intensities were standardized at various concentrations of PpIX (0 to 50  μM) in agarose and alginate tissue phantoms fabricated with ${\mathrm{TiO}}_{2}$ (Titanium IV oxide, 98.0% to 100.5% ${\mathrm{TiO}}_{2}$, ACROS ORG/Thermo Scientific Chemical, #277370010, Haverhill, Massachusetts, United States), hemoglobin (Bovine hemoglobin, blood powder, Sigma Aldrich, #H2625) with optical tissue absorption and scattering properties.^33^^–^^35^ The alginate 3D model was prepared using 2% (w/v) sodium alginate (Alginic acid sodium salt, Sigma Aldrich, #180947) hydrogel phantom crosslinked by $100\;\mathrm{mM}\;{\mathrm{CaCl}}_{2}$ in DPBS (${\mathrm{Ca}}^{2+}$, ${\mathrm{Mg}}^{2+}$ free) solvent as described in earlier studies.^36^^–^^39^

For *in vivo* study, Swiss nu/nu nude mice, aged 6 to 8 weeks and weighing 20 to 25 gms, were obtained from Charles River Laboratories in Waltham, Massachusetts, United States. The animal study protocol was approved by the Institutional Animal Care and Use Committee (IACUC) at UMass Boston, according to the guidelines established by the NIH. Previous studies have shown that this model effectively predicts efficacy in human oral cancer patients.^40^^–^^43^ Prior to implantation, the cells were cultured in a 150 mm cell culture dish (BioLite, Cell Culture-treated Surface, Thermo Scientific, #130183) at 37°C in Ham’s F-12K medium, then trypsinized (trypsin 0.25%) and harvested when cell density reached around $20\times 10^{6}\;\text{cells}/\mathrm{mL}$. Mice were anesthetized using isoflurane inhalation during the subcutaneous implantation. The right flank of each mouse was injected with $6\times 10^{6}$ cells in a 50  μL volume (1:1; Ham’s F-12K, Matrigel^®^ Growth Factor Reduced Basement Membrane Matrix, #356231). Implanted tumors were allowed to grow until they reached ∼5 to 7 mm in diameter (∼65 to $130\;{\mathrm{mm}}^{3}$ in volume at 12 days after implantation). The tumor size was measured by vernier caliper using the ellipsoid volume calculation formula (V=π/6·l·w·h) instead of the modified ellipsoid formula ($V=1/2\cdot l\cdot w^{2}$),^44^ as in our study on resected tumors showed that the height of the tumor (h) is not equal to the width of the tumor (w). On day 12, ALA (200  mg/kg) was injected intratumorally (i.t.) 2.5 h before the light delivery procedure. Prior to PDT treatment and monitoring, mice were anesthetized by isoflurane inhalation. All PDT-treated mice received a fluence of $100\;J/{\mathrm{cm}}^{2}$ at the tissue surface (an irradiance of $70\;\mathrm{mW}/{\mathrm{cm}}^{2}$ over a spot of 1 cm diameter for a duration of 24 min), where real-time monitoring of PDT was based on comparison of tumor site pre-ALA auto-fluorescence, post-ALA PpIX fluorescence, and PpIX photobleaching after the PDT treatment. Furthermore, image-based PpIX fluorescence monitoring was calibrated with a fluorescence spectrometer with a contact mode point measurement probe from Thayer School of Engineering, Dartmouth College, Hanover, NH (see S1 in the Supplementary Material). A total of 14 mice were used in the study. For PDT and subsequent 7-day longitudinal tumor monitoring, four mice were assigned to the control group, which received no treatment, and four mice were assigned to the PDT group. The same two groups were used for post-PDT analysis of necrosis with three mice per group. For histopathological analysis, both control and PDT-treated tumors were extracted 4 days after treatment and embedded in cryo-embedding media (OCT). The embedding process was carried out at low temperatures using dry ice. These sections were stained with hematoxylin and eosin (H&E), and the slides were scanned using an imaging system (Hamamatsu NanoZoomer 2.0-RS, Hamamatsu City, Japan). Image analysis was conducted using Python (see S3 method and Figs. S5 and S6 in the Supplementary Material).

### 2.3 *In Silico* Light Delivery Simulation

Propagation of light through the subcutaneous tumor was modeled *in silico* using the FullMonte software.^45^ FullMonte is a Monte Carlo tetrahedral photon transport simulator software. The simulation geometry consists of two concentric spheres representing a thin layer of skin and a tumor. The skin thickness is taken to be 500  μm, and the tumor diameter is 6 mm based on mouse tumor histopathology analysis. To further model the envisioned clinical use for intraoral light delivery, additional simulations were run based on estimated tissue geometry and optical properties of early-stage oral lesions in human oral epithelium. The tetrahedral mesh is constrained such that no tetrahedron has a volume exceeding $0.1\;{\mathrm{mm}}^{3}$. This value was chosen as a trade-off between accuracy and computational efficiency. FullMonte requires the specification of four optical properties: the scattering coefficient $\mu_{s}$,^46^ the absorption coefficient $\mu_{a}$,^46^ anisotropy g,^47^ and the refractive index η.^47^ These properties are calculated for 630 nm wavelength, in accordance with that used in the experiment, and are tabulated in (see Table S1 in the Supplementary Material). The light source is simulated as a cut-end fiber, such that the beam spot has a diameter of 1 cm, in accordance with *in vivo* experimental estimates. The absorbed photon weight per tetrahedral provided as the output of Fullmonte is converted to physical PDT units (${J/\mathrm{cm}}^{2}$) using the absorption coefficient and the prior photon packages to joule calibration.^48^

### 2.4 Statistical Analysis

The significant difference in central values was evaluated using R (Comprehensive R Archive Network)^49^ with installed statistical analysis packages. The *in vitro* input parameters used for the statistical significance evaluations are PpIX fluorescence, auto- and photobleaching fluorescence characteristics, and linear correlation between ${\mathrm{TiO}}_{2}$ phantom with or without hemoglobin. For *in vivo* studies, parameters are PpIX fluorescence on tumor surface, post-PDT necrosis as z-depth, and area necrosis post-PDT after image segmentation using Python OpenCV packages.^50^ The significant and nonsignificant differences in the means were assessed using the Kruskal–Wallis test, which is a nonparametric alternative to one-way ANOVA. Significance levels were set as the convention of p-values: ***p<0.001; **p<0.01; *p<0.05.

## 3 Results and Discussion

### 3.1 *In Vitro* Device Performance Evaluation

**Fig. 2:**
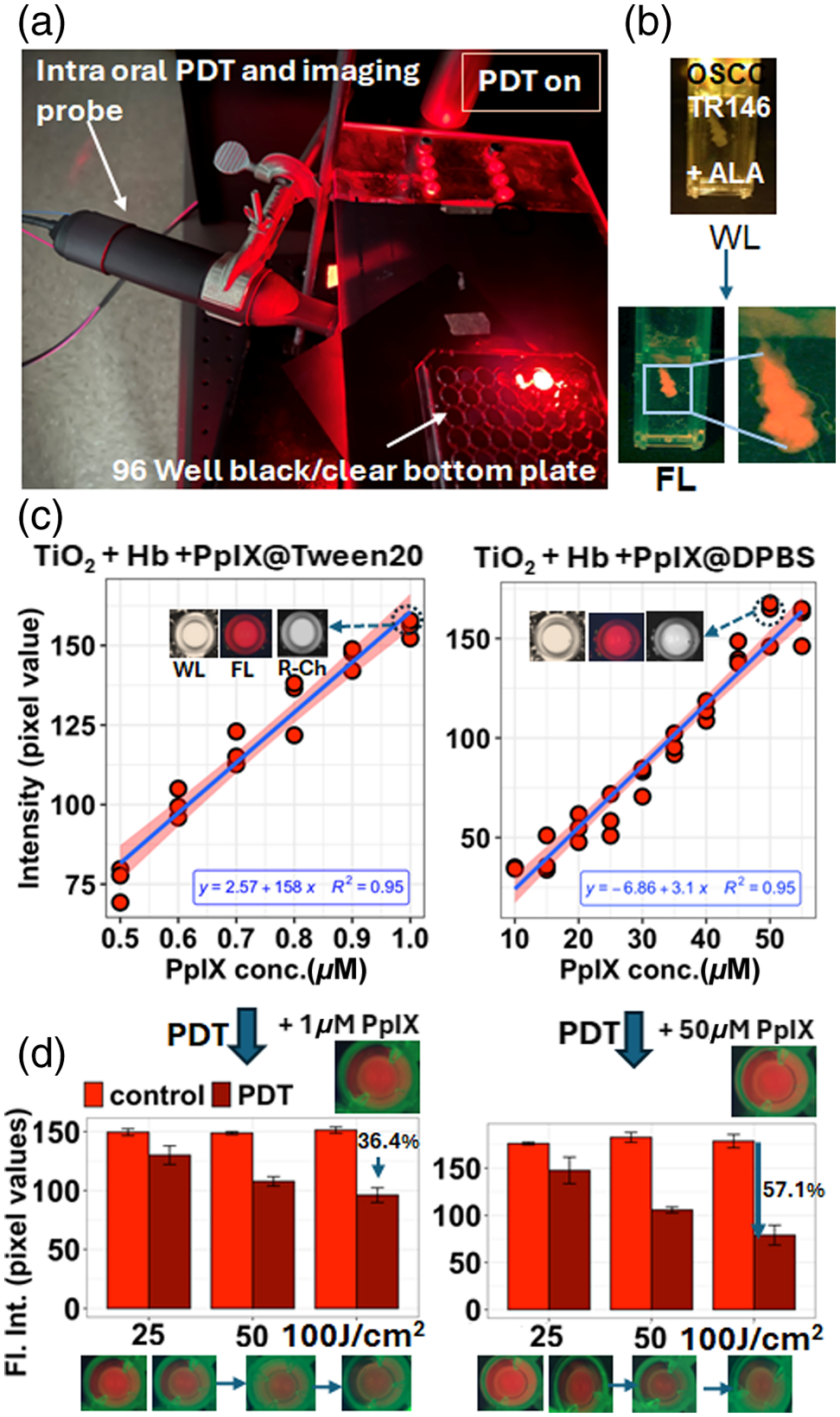
(a) PpIX imaging and PDT dosimetry in 96-well plate tissue phantoms. (b) Fluorescence imaging captured the ALA-photosensitized OSCC TR146 aggregates within the alginate phantom in a disposable PMMA cuvette. (c) The 96 wells’ PpIX fluorescence intensity quantification exhibited a linear correlation ($R^{2}=0.95$) with PpIX concentration in the ${\mathrm{TiO}}_{2}$ (0.5 g/L) + hemoglobin (Hb; 1.0  g/L) phantom with and without Tween20 surfactant (0.01%). (d) Post-PDT dosimetry revealed a reduction in PpIX (1 and 50  μM) fluorescence intensity (36.4% and 57.1%) due to photobleaching caused by a light dose of $100\;J/{\mathrm{cm}}^{2}$ PDT.

The imaging capabilities of the integrated device were initially evaluated using tissue phantoms (a liquid form) with a range of PpIX concentrations. For these measurements, the intraoral probe was held above or below the phantom samples in 96-well plates at a fixed distance (1 cm) during imaging and PDT treatment procedures, as shown in Fig. 2(a). Homogenous tissue phantoms were prepared by mixing PpIX at specified concentrations (0.5 to 50  μM) with titanium dioxide (${\mathrm{TiO}}_{2}$; 0.5  g/L, $\mu_{s}@630\;\mathrm{nm}=9.4\;{\mathrm{cm}}^{-1}$) as a standard scattering agent and hemoglobin (Hb; 1.0  g/dL, $\mu_{a}@630\;\mathrm{nm}=1.2\;{\mathrm{cm}}^{-1}$) as an absorption agent.^34^^,^^51^^,^^52^ Here, specified PpIX concentrations are based on physiological PpIX where PpIX in the normal human body have been reported to be in the range of 2 nM in blood plasma^53^ and in normal human tissue below 0.2  μM,^54^^,^^55^ wherein the tumor tissue after ALA administration is in the range of 0.2 to 50  μM^56^ along with high blood plasma PpIX (0.06  μM) after 4.6 h post-ALA.^53^ The linearity of the fluorescence signal was established in PpIX solutions in DPBS as well as in DPBS/Tween-20 (0.01%)^57^^,^^58^ [Fig. 2(c)].

PpIX is a hydrophobic porphyrin compound, and its physiological state in cancer cells is influenced by the intercellular accumulation that occurs after the exogenous application of ALA. PpIX tends to aggregate in the hydrophilic cytoplasm; however, this aggregation dissociates upon interaction with the lipid membrane, which in turn enhances the fluorescence intensity of PpIX. In a previous study, it was observed that different types of cancer cells showed accumulation of PpIX in the perinuclear space of the lipid membrane 4 h after ALA application. This accumulation translocated to cellular lipid membrane after 24 h, resulting in greater concentrations of PpIX at the cellular lipid membrane compared to perinuclear space.^59^ Similarly, in our study, TR146 showed strong PpIX fluorescence at 4 h, and the intraoral device was able to detect PpIX fluorescence in cell culture aggregates of TR146 (6 to 10 million cells) that had been incubated with ALA (3 mM), resulting in the production of endogenous PpIX (1.5  μM/μg of cellular protein) compared to control samples [Fig. 2(b)]. In both DPBS and DPBS/Tween-20 solvents, PpIX fluorescence intensity captured by our intraoral device showed linear ($R^{2}=0.95$) dependence over a range of PpIX concentrations (0.5 to 1.0  μM; in Tween-20 + Hb, 10 to 50  μM; in DPBS + Hb). An important capability of the integrated device is the ability to measure PpIX photobleaching, which is the decrease in fluorescence due to the degradation of fluorophores during light exposure. In PDT, the extent of photobleaching has been leveraged as a surrogate reporter of dose deposited in tissue.^60^ This is an important feature of the integrated device for monitoring PDT treatment, potentially in real time going forward. The use of photobleaching as a dose reporter to increase the probability of complete response is motivated by our clinical results showing a decisive correlation (p<0.001) between outcome and PpIX bleaching at the time of treatment.^18^^,^^28^ Notably, after a dose of $100\;J/{\mathrm{cm}}^{2}$ at an irradiance of 33 to $54\;\mathrm{mW}/{\mathrm{cm}}^{2}$, a 40% to 50% reduction in photobleaching was observed among patients who responded successfully to PDT. Likewise, in liquid tissue phantoms, photobleaching scales with dose as seen in phantoms exposed to an irradiance of $70\;\mathrm{mW}/{\mathrm{cm}}^{2}$. Photobleaching was reported by 36.4% and 57.1% (mean values) in Tween-20 and DPBS media phantoms, respectively [Fig. 2(d)], aligning well with previous clinical results.

**Fig. 3:**
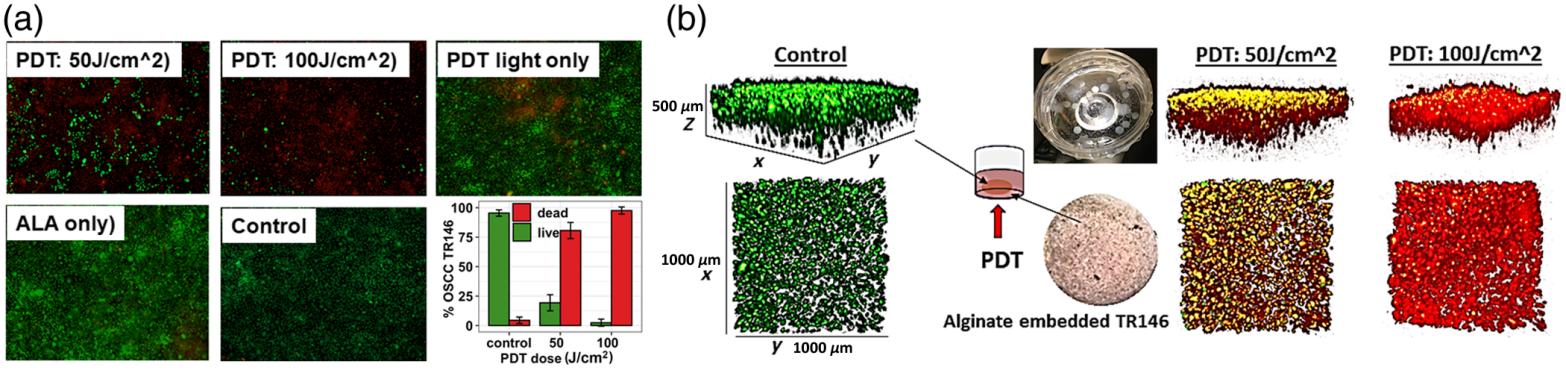
(a) PDT treatment of TR146 cell cultures photosensitized with ALA using the intraoral device. The subpanel shows representative images of monolayer cultures treated with a total light dose from the device of 50 and $100\;J/{\mathrm{cm}}^{2}$ and stained with calcein AM and ethidium bromide, labeling live cells green and dead cells red, respectively. The bar graph represents the cell killing with increased light dose. (b) The Zeiss LSM 880 confocal fluorescence imaging of alginate-${\mathrm{TiO}}_{2}$ phantom entrapped TR146 showed in-depth (z-axis) increased cell killing with increased PDT doses.

In further evaluation, we examined the use of the intraoral probe for fluorescence imaging and monitoring of PDT treatment in tissue phantoms containing TR146 oral squamous cell carcinoma (OSCC) cells. We initially applied increasing doses of PDT to assess ALA-PDT-induced necrosis in a 2D monolayer (see Fig. S7 in Supplementary Material). Using calcein AM and EtBr fluorescence probes, we quantified live and dead cells compared with controls through image segmentation analysis with Python OpenCV Toolbox (see S4 in the Supplementary Material). The segmentation analysis revealed almost 95% cell death [Fig. 3(a)]. Subsequently, we evaluated the same PDT parameters in TR146 3D cell cultures (∼2  mm spherical shapes), which better mimic human disease, particularly lesions in the oral epithelium (lesion epithelium thickness ∼600 to 900  μm).^61^^,^^62^ These cultures were created by suspending TR146 cells in alginate hydrogels [Fig. 3(b)] with a scattering agent ${\mathrm{TiO}}_{2}$. Here, a high-density cell aggregate (7.5 million cells) was prepared from TR146 cells photosensitized with ALA, then after 4 h, aggregated and mixed with ${\mathrm{TiO}}_{2}$ to simulate tissue scattering. After PDT treatment, confocal imaging (Zeiss LSM 880) was used to assess the cytotoxic response [Fig. 3(b)].

**Fig. 4:**
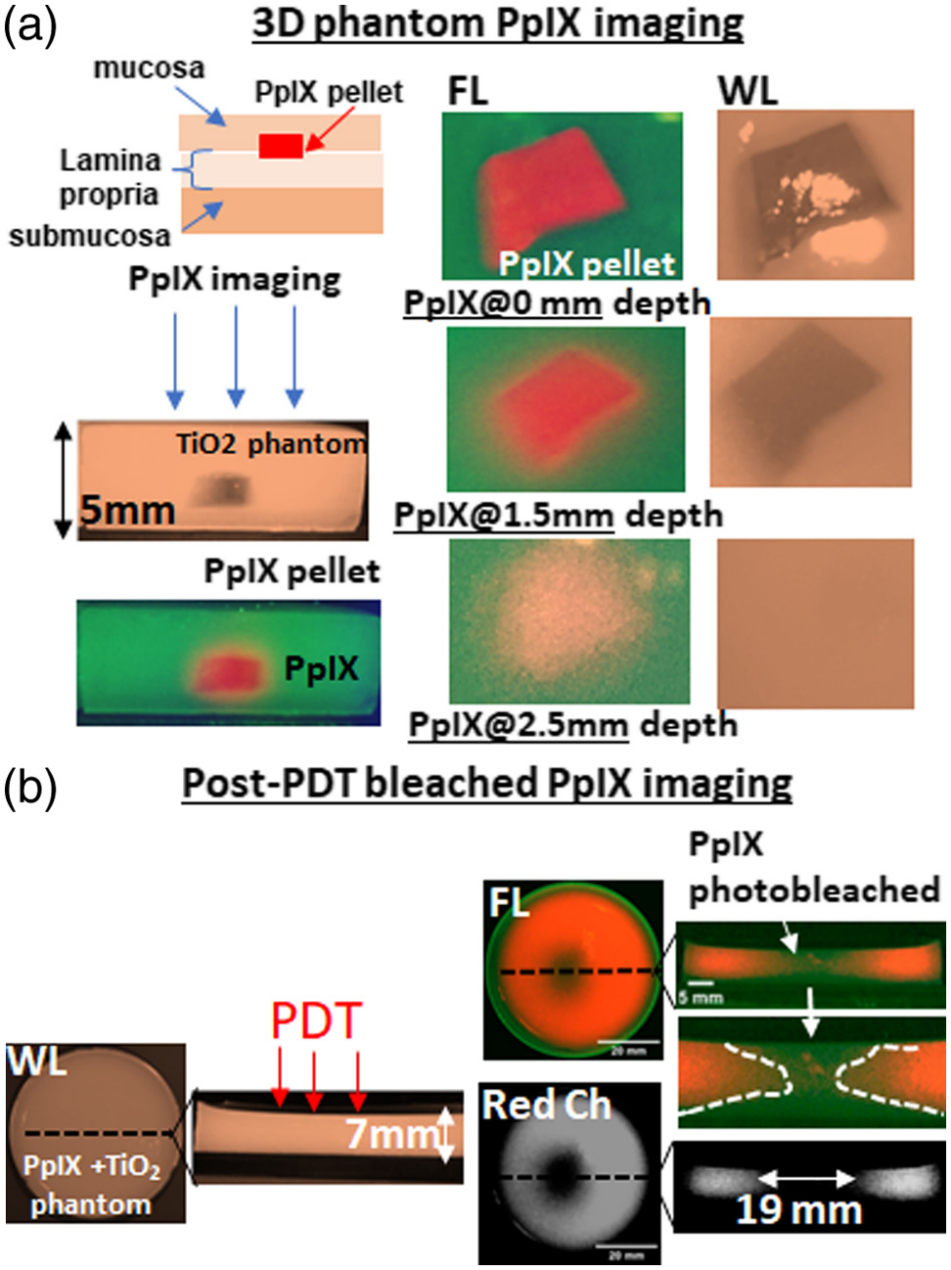
PpIX imaging of buccal mucosa tissue models of CIS and lamina propria micro-invasive OSCC. (a) PpIX pellet (50  μM) was imaged up to 2.5 mm depth in agarose + collagen + ${\mathrm{TiO}}_{2}$ phantom. (b) PpIX photo-bleaching on the surface as well as in the tissue phantom after device-mediated $100\;J/{\mathrm{cm}}^{2}$ PDT dose.

To determine the depth of PDT response, the buccal mucosal epithelial phantom was prepared with collagen (5  mg/mL)^38^ and ${\mathrm{TiO}}_{2}$ (0.5  g/L) in agarose (1.5%). A 50  μM PpIX agarose block was placed at up to 2.5 mm (or 2500  μm) depth, and fluorescence and WL imaging were taken, placing the probe above the phantom 1 cm [Fig. 4(a)]. The device is able to capture the PpIX fluorescence up to 2.5 mm depth after 405 nm LED light excitation. Surface photobleaching following PDT ($100\;J/{\mathrm{cm}}^{2}$) was observed in fluorescence imaging [Fig. 4(b)]. We also used the device to assess the depth of photobleaching by physically cutting and imaging the cross-section view of tissue phantoms. The ∼7  mm depth of photobleaching observed is consistent with expectations for such an optically opaque phantom. With the addition of hemoglobin (1.0  g/dL), the depth of photobleaching was 2.6 mm (see Fig. S4 in the Supplementary Material), which may be relevant in cases with some extent of vascularity, for example, in premalignant (erythroplakia) and micro-invasive malignant lesions.

**Fig. 5:**
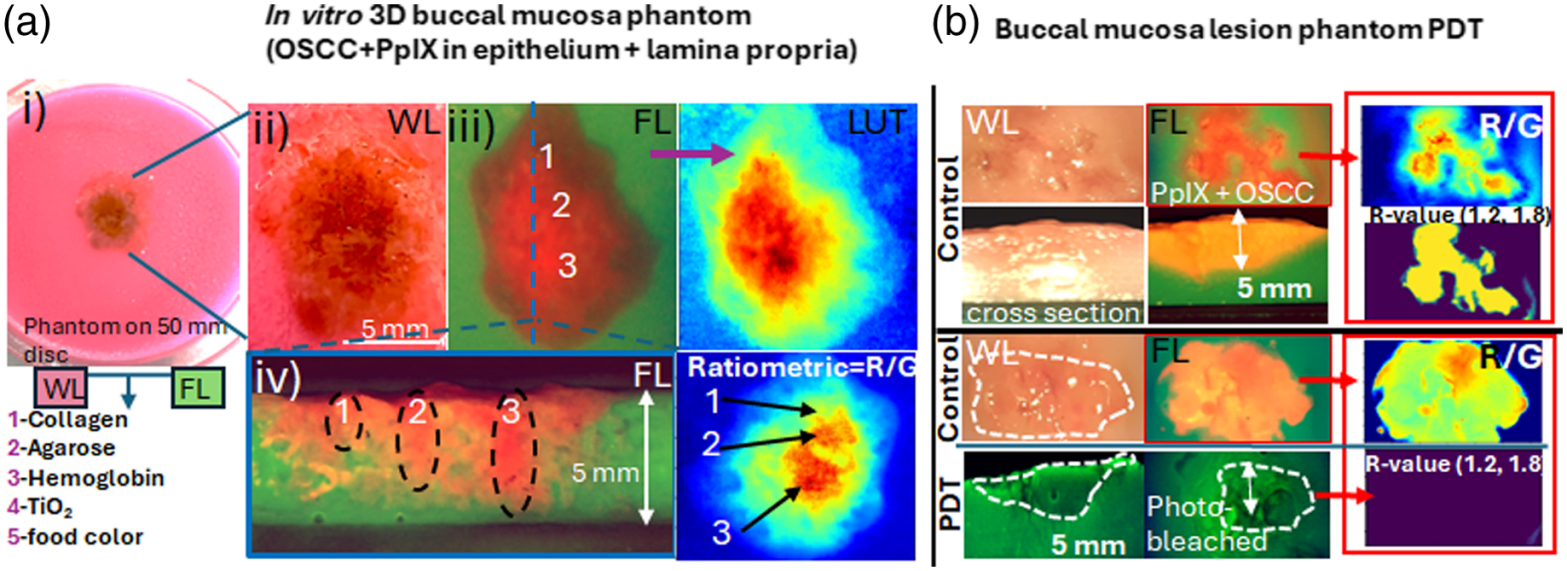
*In vitro* model of oral lesions with PpIX photosensitization simulates the buccal mucosa up to a length of 5 mm. This includes the epithelium and lamina propria (a). ALA photosensitized OSCC TR146 was embedded in a phantom made with collagen, hemoglobin, and ${\mathrm{TiO}}_{2}$ to mimic clinically relevant tissue autofluorescence, scattering, and absorption. Ratiometric imaging shows the 1, 2, and 3 high Rv (ratiometric) regions with depth of OSCC-PpIX accumulation, and margins of the OSCC lesion (b). PDT in buccal mucosal phantoms with imaging of PpIX fluorescence using ratiometric-value ranges (Rv).

Furthermore, to simulate lesions in the oral mucosa, tumor cell aggregates (mean value: 7.5 million TR146 cells) were embedded in a phantom made of collagen (5  mg/mL), agarose (1.5%), hemoglobin (0.01  g/dL, $\mu_{a}@630=0.2\;{\mathrm{cm}}^{-1}$), and ${\mathrm{TiO}}_{2}$ (0.5  g/L) at various depths [see Fig. 5(a)]. The variation of OSCC cell depth is motivated by modeling field cancerization in the oral cavity [Slaughter’s concept: hyperplasia → low-grade dysplasia; (i) mild dysplasia, (ii) moderate dysplasia → high-grade dysplasia; (i) severe dysplasia, (ii) carcinoma *in situ* (CIS) → invasive SCC].^63^^,^^64^ Here, the extent of PpIX production in OSCC (severe dysplasia) is higher compared with dysplastic cells, but it was reported that the PpIX production shows a contrast in the field of cancerization compared with adjacent normal cells.^65^^,^^66^ Cell aggregates were placed within 5 mm (5000  μm) of the tissue phantom surface to simulate an oral epithelial malignant tumor breaching the basal layer of the oral mucosa to invade the lamina propria. The thickness of buccal mucosal epithelium with dysplasia is ∼900  μm, compared with a normal epithelium thickness of 500  μm.^61^^,^^62^ Various dysplastic stages, such as mild dysplasia, moderate dysplasia, and severe dysplasia, extend to approximately one-third, two-thirds, and more than two-thirds of the epithelium thickness from the basal layer, respectively.^67^

In this study, we incorporate a reconstituted collagen ECM in the phantom, using it as a physiologically relevant source of tissue autofluorescence. In tissue, metabolic coenzymes such as flavin adenine dinucleotide (FAD) and nicotinamide adenine dinucleotide (NADH) reflect the metabolic activities of squamous epithelial cells and serve as markers for various stages of dysplasia alongside the signal from collagen.^68^ When normal tissue, consisting of the epithelium and lamina propria, is exposed to light with wavelengths between 300 and 450 nm, it exhibits autofluorescence attributed to NADH and FAD in the epithelium, as well as collagen in the lamina propria.^69^ In cases of epithelial dysplasia and invasive OSCC, the tissue matrices within the lamina propria show a disorganized and loose collagen structure^70^ resulting in lower auto-fluorescence.^68^^,^^69^ It was also reported that collagen in the matrix of epithelial dysplasia also shows disorganized collagen that is absent in normal epithelium.^71^^–^^73^

Here, we use a relatively low hemoglobin concentration in oral tissue phantoms as it has been reported to be negligible in cases of normal and mild epithelial dysplasia. In moderate to severe dysplasia conditions, immunohistopathology biomarkers indicate increased vascularity beneath the epithelium in the papillary regions^74^ and angiogenic shift in the connective tissue in lamina propria. In practice, this vascularity obstructs 405 nm light during PpIX imaging primarily due to absorption by hemoglobin.^75^

**Fig. 6:**
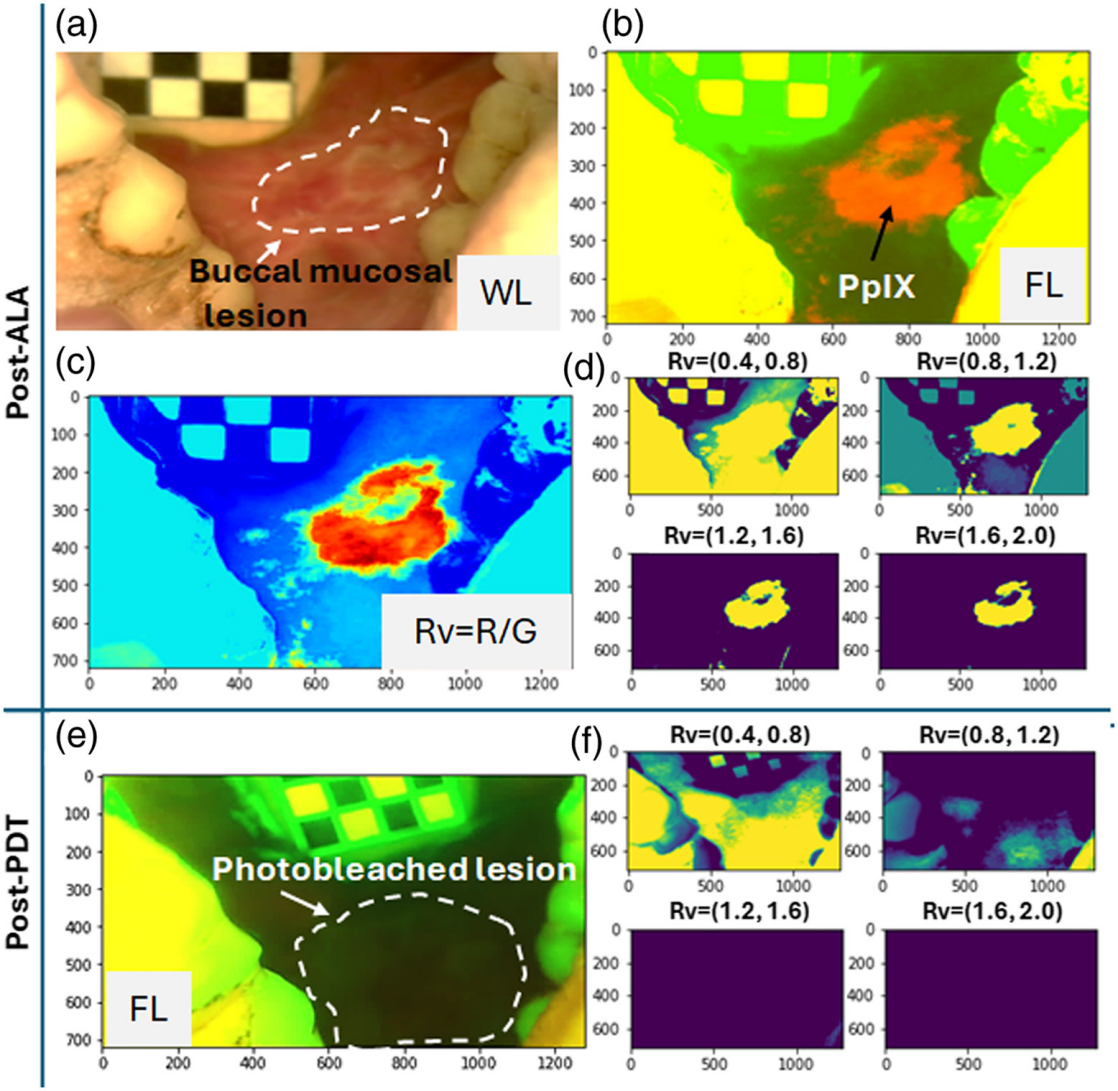
Clinical fluorescence imaging of buccal mucosa lesion using the prototype of the integrated intra-oral device with existing imaging components. (a), (b) WL and FL imaging of oral lesion near retro-molar trigone (RT) after photosensitization with PpIX. (c), (d) The ratiometric imaging of lesion site PpIX with various combinations of ratiometric-value (Rv) ranges.

Following our previous clinical oral cancer PDT study,^28^ we analyzed the ratiometric values of red and green fluorescence in relation to lesion parameters (specifically PpIX photosensitized OSCC) within the tumor phantom [see Fig. 5(a)]. We compared the tissue phantom’s ratiometric values (Rv) to those of malignant lesions in clinical oral cancer patients (as shown in Fig. 6). In our earlier study, we established a threshold Rv range of 1.2 to 1.8 based on observations made during pre-ALA, post-ALA, and post-PDT treatments,^28^ which significantly correlated with ultrasound (US) findings. Previous research has indicated that the relative fluorescence intensity in the split channels (red, green, blue) can effectively distinguish between malignant and normal tissues.^76^^,^^77^ Sharwani et al.^78^ demonstrated the capacity to differentiate malignant tissue from various stages of oral premalignant lesions, including dysplasia, hyperplastic tissue, and inflammation. Their findings showed that dysplastic tissue and carcinoma *in situ* (CIS) exhibited higher Rv values, achieving 83% to 90% sensitivities and specificities of 79% to 89%. We monitored and quantified PDT light-mediated photobleaching in a clinical study utilizing the imaging capabilities of an intraoral device.^28^ In this study, we integrated the PDT features into the existing intraoral device. The results on the phantom revealed surface photobleaching and a region of photobleaching at a depth of 5 mm. Ratiometric image analysis indicated that the Rv values fall within the range observed in our clinical studies [Fig. 5(b)].

### 3.2 *In Vivo* Device Performance Evaluation in Murine Tumor Xenografts

**Fig. 7:**
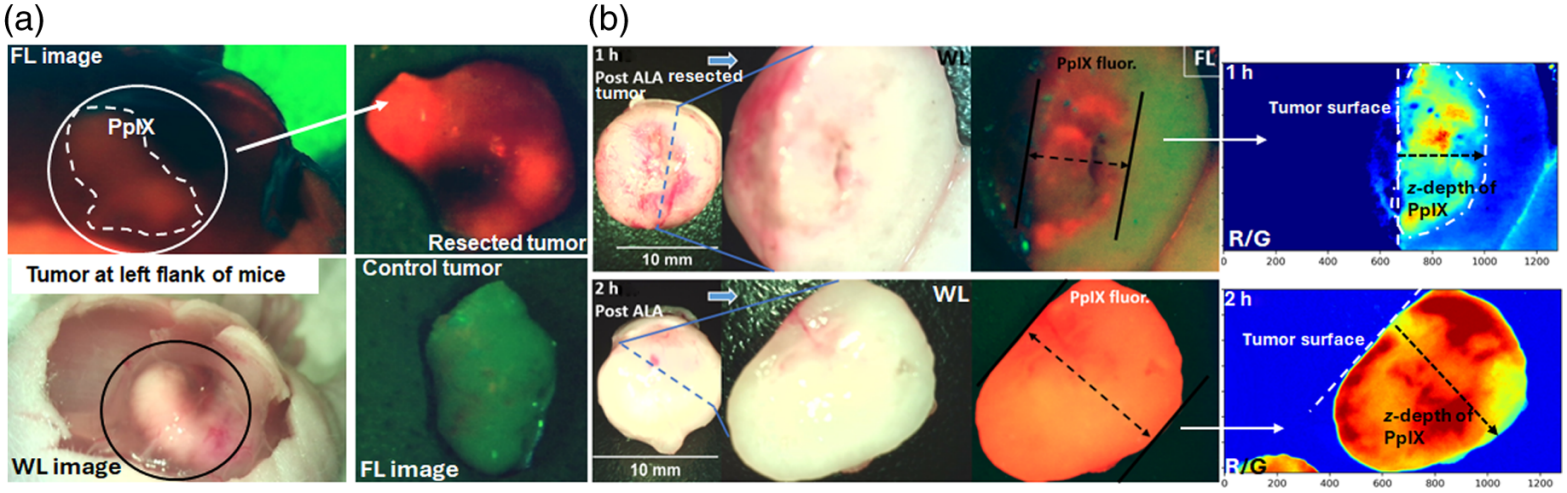
Time-dependent intratumoral PpIX accumulation. (a) The tumor, sensitized by injection of ALA, was exposed after removing the skin flap on the tumor surface. A device-mediated fluorescence (FL) image was compared with the control resected tumor. (b) Fluorescence (FL) and ratiometric analysis of the half-cut tumor shows the extent of PpIX accumulation 1 and 2 h after ALA administration.

**Fig. 8:**
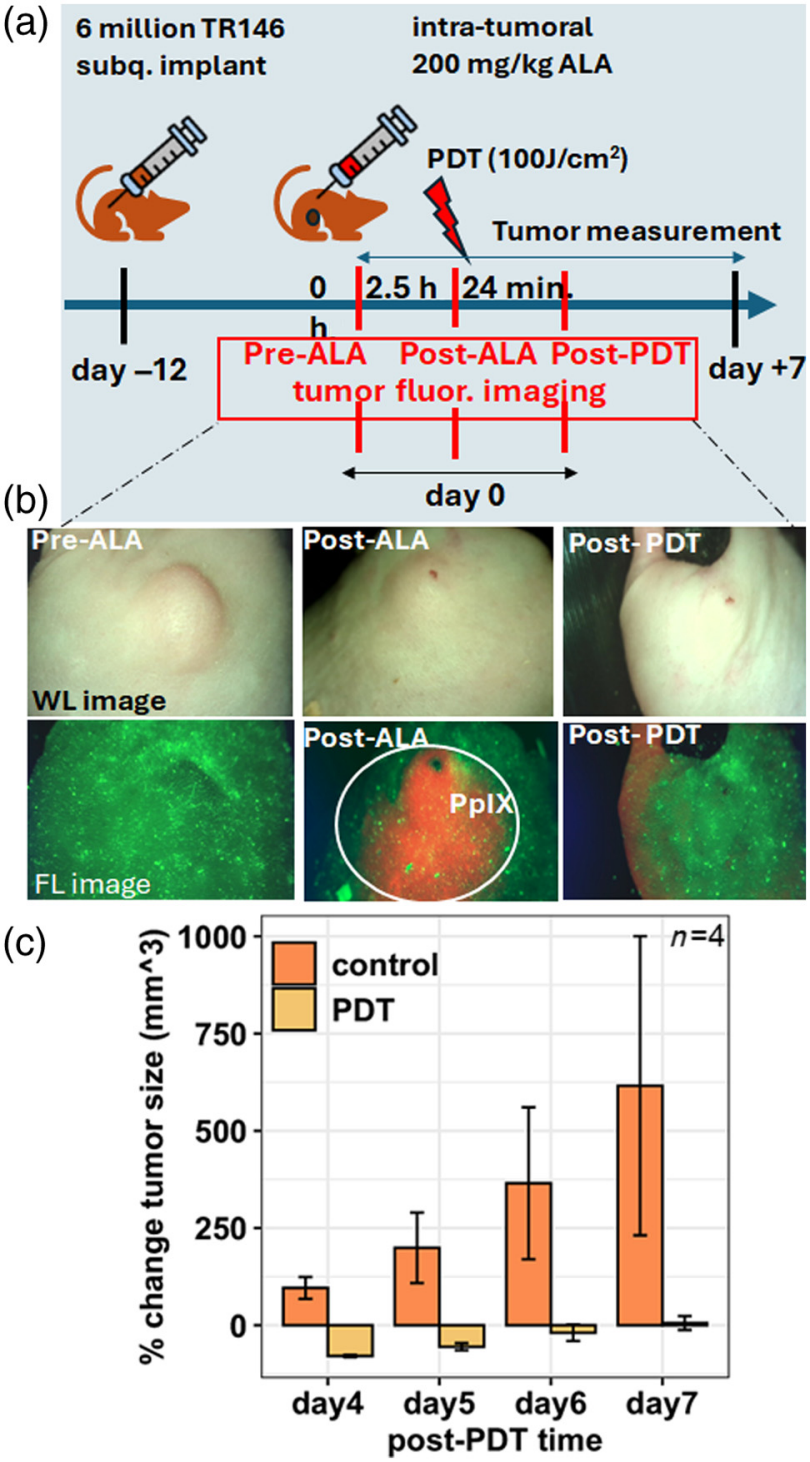
*In vivo* intraoral PDT in subcutaneous OSCC mouse model. The timeline (a) shows mice were implanted with TR146 cells, forming mature tumors 5 to 7 mm in diameter over 12 d before PDT treatment. PDT-treated mice were photosensitized by intra-tumoral injection of ALA. (b) Fluorescence emission shifts from green to red before to after ALA injection and localized photosensitization in the same animals. (c) Longitudinal volume measurements at time points up to 7 d post-treatment.

After establishing key performance parameters *in vitro*, we proceeded with further integrated device assessments using mouse tumor xenografts (see Figs. 7 and 8). The mouse subcutaneous tumor model did not represent the actual anatomical tissue of early oral epithelium lesions (either dysplastic or CIS OSCC). The subcutaneous model tissue is much thicker than the 900  μm to 1.2 mm thick oral epithelium lesion. If the lesion epithelium is highly vascularized and fibrous or extends beyond the lamina propria after breaching the basal layer, it may represent the same thickness as the mouse tumor model. In these studies, multichannel fluorescence imaging proved essential for visualizing OSCC tumor tissues in live animals, particularly due to the significant autofluorescence background. Immune-compromised athymic Swiss Nu/nu mice were utilized for these experiments^41^^,^^42^ as it was reported that the subcutaneous model is effective for head and neck SCC PDT.^79^ OSCC cells were cultured, harvested, and then implanted subcutaneously ($6\times 10^{6}$ cells) into the flank of each mouse. The tumors were allowed to grow for 12 days before ALA administration, imaging, and PDT treatment^42^ [see Fig. 8(a) timeline]. Here, ALA (200  mg/kg) was administered intratumorally. In previous studies, ALA has been injected intravenously (250 to 500  mg/kg),^19^ but with increased off-target toxicity, including skin accumulation and post-PDT skin phototoxicity in the irradiated skin covering the subcutaneous tumors.^80^ The intratumoral route for the delivery of photosensitizers more broadly has also been a subject of renewed interest.^81^ The pharmacokinetics of PpIX production in post-ALA (200  mg/mL) subcutaneous tumors were analyzed to determine the optimal photosensitization time (conversion of ALA to PpIX) for PpIX accumulation throughout the entire tumor. For these experiments, the skin was surgically retracted from the tumor, allowing us to visualize PpIX accumulation within the tumor. The fluorescence imaging, taken 1-h post-ALA injection, indicated that PpIX production was present in approximately half of the intratumoral space. By contrast, 2 h after ALA injection, PpIX was distributed more uniformly throughout tumors [see Fig. 7(b)]. Based on these initial results on the resected tumor (nearly 10 mm in size), we standardized the post-ALA time up to 2.5 h for optimum PpIX photosensitization in this model. Further comparative analysis was carried out on tumors resected from animals that had not received ALA injection, confirming that the fluorescence signal is predominantly in the green spectral window of the intraoral camera when in the absence of ALA photosensitization. This photosensitization process was faster than previously reported with intravenous^42^ and intraperitoneal routes,^82^ which typically require 4 h for effective photosensitization of subcutaneous murine tumors. In addition, ratiometric imaging using the intraoral device highlighted the depth of photosensitization achieved at the 1-h and 2-h mark following ALA administration [Fig. 7(b)].

The integrated device’s ability to longitudinally image animals was evaluated, where confirmed photosensitization after ALA injection was compared to before. In addition, expected photobleaching after light delivery to the target tumor was assessed [see Figs. 8(b) and 8(c)]. The endogenous autofluorescence (green fluorescence) signal on the skin above the tumor and surroundings was dominant before ALA injection [Fig. 8(b)]. After injecting ALA [Fig. 8(b)], the red fluorescence signal becomes dominant from the conversion of ALA into PpIX corresponding to photosensitization. In clinical implementation, the ability to monitor the progress of photosensitization is important to confirm PpIX accumulation prior to light delivery.

**Fig. 9:**
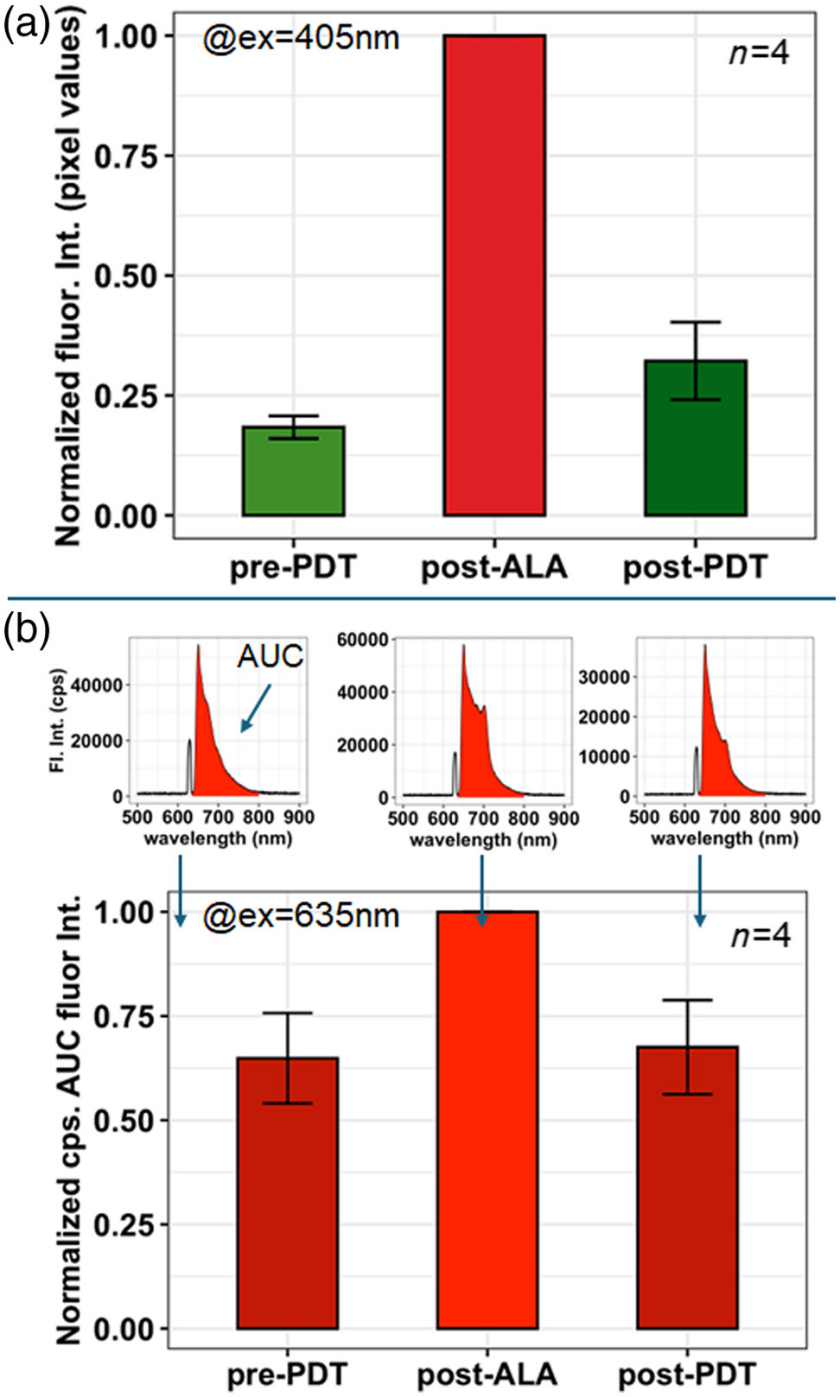
Comparison of tissue surface fluorescence imaging using blue-violet excitation from the intraoral device versus fluorescence spectroscopy measurement of the same tissue using red-light excitation of PpIX. (a) Red channel fluorescence intensity was measured after excitation of the tumor with 405 nm light. (b) The fluorescence area under the curve (AUC) was quantified in the range of 635 to 680 nm after the excitation of the tumor with 635 nm laser light.

It could be used with automated image analysis to provide a simple go/no-go indicator to nonexpert users that it is time to commence with treatment. Here, post ALA, red channel image processing showed PpIX fluorescence intensity on the skin above the tumor increased to ∼80% after 2.5 h [Fig. 9(a)]. Post-PDT analysis showed the photobleaching up to ∼70%. These results are consistent with our previous clinical study using the prototype of the intraoral device with only imaging property, where fluorescence imaging showed the spike in buccal mucosal lesion site PpIX fluorescence increased to ∼62%, and post-PDT PpIX photobleached to ∼50%.^28^ In the clinical study, the extent of photobleaching in PDT-treated oral lesions correlates strongly with the outcome, underscoring the utility of being able to monitor photobleaching during treatment. Post-ALA and post-PDT image analysis showed a significant decrease in skin surface autofluorescence compared with pre-ALA. Recognizing that PpIX photobleaching measurements using blue-violet (Soret-band) excitation are limited to the tissue surface, we sought to independently measure PpIX fluorescence in mouse tumors using a spectroscopic measurement device,^83^ which uses a 635 nm laser source for excitation [corresponding to the Q4 absorption band, Fig. 1(b)] (see S1 and Fig. S1 in the Supplementary Material). Using red light (∼635  nm) excitation, we estimate that fluorescence from up to ∼5  mm depth is probed, in comparison to less than 1 mm using the oral probe.^51^^,^^84^^,^^85^ Here, we use the spectroscopic device to measure total PpIX fluorescence intensity in the range of 635 to 680 nm (AUC) at three time points: pre-ALA, post-ALA, and post-PDT [see Fig. 9(b)]. The PpIX measurements indicated that post-PDT bleaching was significant. Moreover, the photosensitization quantified by the dosimetry device was 35% higher, whereas the superficial photosensitization measured with the intraoral device was 82% higher compared with the pre-ALA measurements. The post-PDT PpIX bleaching observed by the dosimetry device was 32%, and by the intraoral device, it was 68%. Overall, these results show that surface photobleaching is correlated with deeper tissue bleaching, though the extent of change in PpIX signal upon photosensitization and photobleaching is more dramatic. This is likely because the absorption efficiency in the Soret band is far higher than the Q4 band of PpIX, resulting in a higher fluorescence signal, even though it is limited to a smaller excitation volume.

**Fig. 10:**
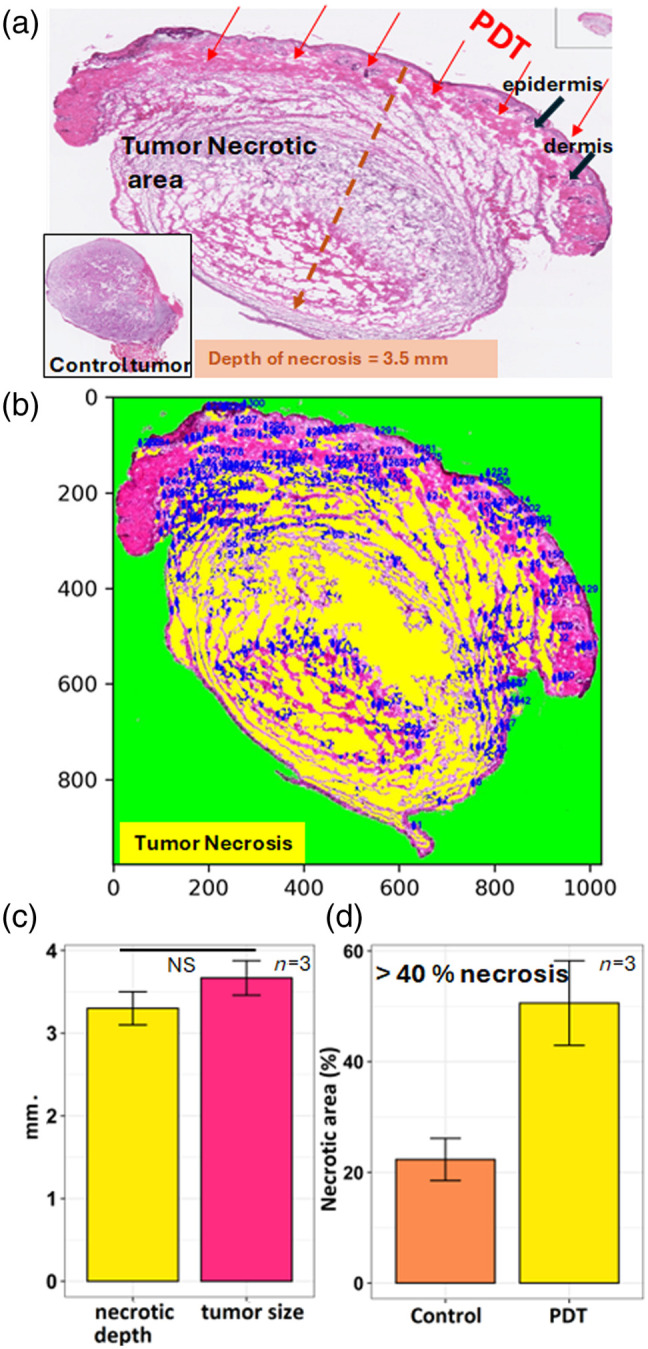
Histopathology assessment of PDT-induced necrosis in tumors resected 4 d after treatment. (a) The necrotic area of the tumor is visible beneath the healthy skin (epidermis and dermis) up to a depth of 3.5 mm. (b) Tumor necrotic area quantification is marked as yellow segmented color. (c) The depth of necrosis as compared to the size of the tumor (short-axis of ellipsoid; in the direction of PDT light). (d) The extent of tumor necrosis >40% post PDT.

To evaluate the response of PDT delivered through an intraoral device, mice underwent longitudinal tumor measurements using a digital caliper over a period of up to 7 days [see Fig. 8(c)]. Following PDT treatment (1 to 3 days), the tumors exhibited an inflammatory response/edema, making it difficult to accurately measure tumor size with a digital caliper. Edema was observed, along with the skin surrounding the tumor turning a dark-reddish color; however, this edema subsided after 3 days. This is attributed to partial vascular damage and significant destruction of OSCC.^42^^,^^86^ Notably, post-PDT treatment at a dose of $100\;J/{\mathrm{cm}}^{2}$ (at $70\;\mathrm{mW}/{\mathrm{cm}}^{2}$) resulted in a significant tumor response by day 4 compared with the control (nontreated) tumors. This significant difference persisted up to day 7, during which the size of the control tumors increased exponentially [Fig. 8(c)]. Post-PDT analysis of intra-tumoral necrosis was conducted using separate sets of mice. According to clinical and *in vivo* studies, the extent of necrosis serves as a bellwether of successful PDT.^18^^,^^42^^,^^87^ Imaging of the H&E sections reveals a necrosis depth of up to 3.3 mm (mean value) (Fig. 10), affecting the entire tumor area while leaving the dermis and epidermis intact, indicating a complete accumulation of ALA in the tumor without affecting the surrounding healthy tissue. Image segmentation quantification indicates that necrosis accounted for more than 40% of the tumor area compared with the control group [Figs. 10(b) and 10(d)]. In our previous clinical study, ALA-PDT achieved a maximum depth of necrosis of 7.2 mm in oral cancer lesions by applying the same PDT regimen.^18^ The depth of necrosis enhancement was attributed to possible photodynamic priming (PDP)–mediated effector T cell induction, which is not present in the athymic nude mice used here.^88^

**Fig. 11:**
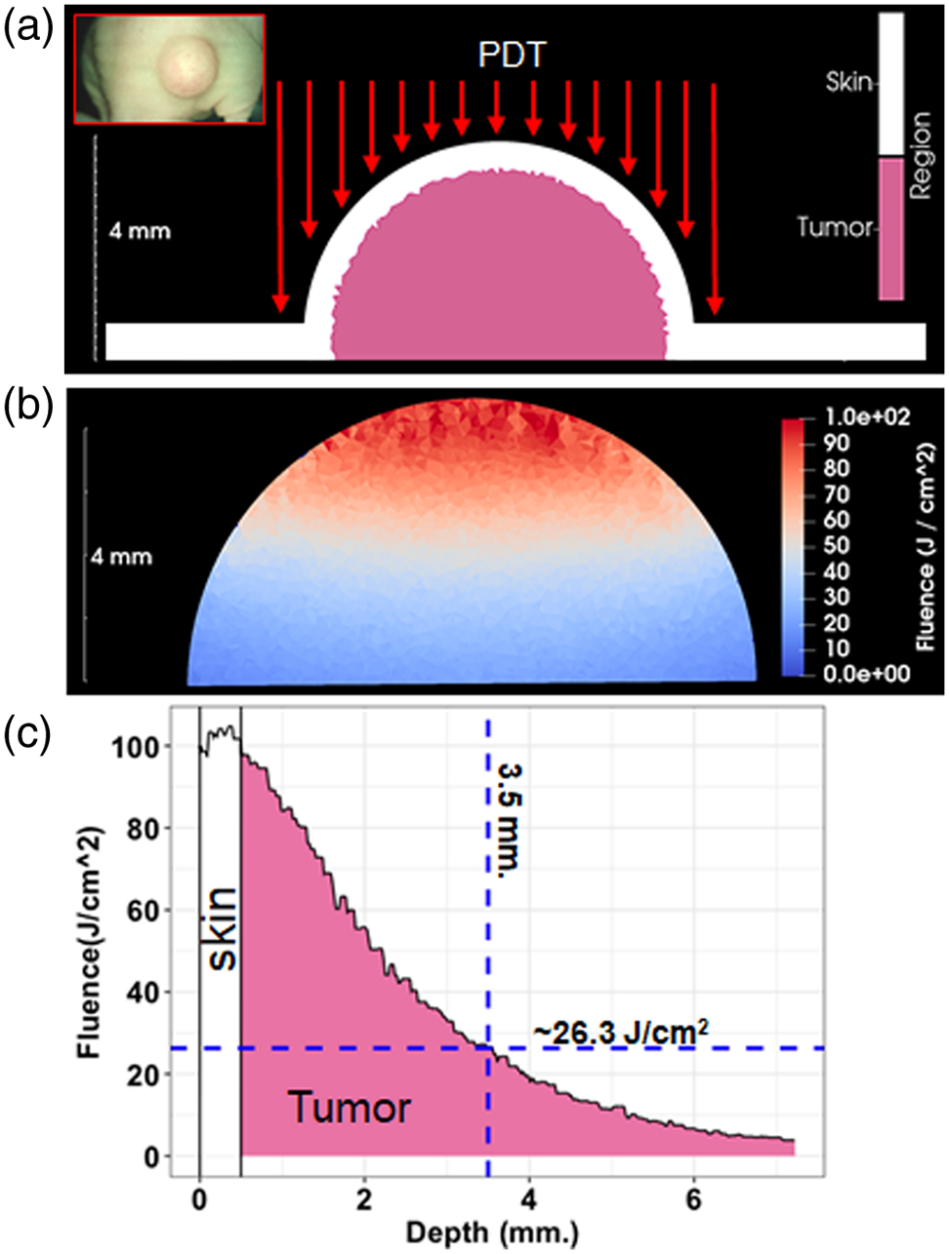
Modeling fluence deposited during transdermal light delivery to subcutaneous tumor xenografts. (a) The light propagation model uses a modified spherical geometry similar to the *in vivo* TR146 subcutaneous tumors (inset image). (b) Heat-map of PDT light fluence. (c) Fluence as a function of depth (c).

The FullMonte PDT light dosimetry package^45^ was utilized to simulate light propagation in nude mice with subcutaneous tumors and estimate the effective fluence at various depths (Fig. 11). Previously, FullMonte Monte Carlo simulation was successfully implemented in clinical and preclinical PDT to investigate light propagation in various tissues and tissue phantoms.^89^^,^^90^ Here, a cut-end fiber was used as a light source, with a specified number of photons absorbed in a 3D volume modeled as a tetrahedral mesh structure, representing *in vivo* tissue. The optical properties of the skin (∼500  μm thickness)^91^ and subcutaneous tumor tissue of nude mice at 630 nm were incorporated into the simulation^46^^,^^47^ (see Table S1 in the Supplementary Material). Overall, the estimated dependence of fluence on tissue depth is aligned with the histopathologic examination of *ex vivo* PDT-treated tumors, in which we see that the depth of necrosis correlates with the depth at which a minimum fluence of $∼26.3\;J/{\mathrm{cm}}^{2}$ was deposited [Figs. 11(b) and 11(c)].

Although the simulation presented above was designed to model the experimental scenario of the present study, these implanted tumors in mice are likely to be thicker than the early-stage lesions, which the image-guided PDT platform is intended to treat. To model light delivery that more closely recapitulates the envisioned clinical scenario, we conducted additional FullMonte MC simulations modeling of early oral lesions (CIS or dysplasia), which have not penetrated the basal layer.^67^ In this simulation, the oral mucosa parameters are derived from previous clinical studies.^18^^,^^62^^,^^92^^,^^93^ The optical parameters were taken from earlier findings^51^^,^^94^ on oral lesions tabulated in the Supplementary Materials and methods (Fig. S2, method S2 and Table S2 in the Supplementary Material). The layers consist of a thin layer of saliva, an upper epithelial layer without tumor cells, an epithelial layer containing atypical/tumor cells, the basal layer, and the lamina propria, with a combined thickness of up to 2.3 mm, as illustrated in Fig. S3 in the Supplementary Material. The thin saliva layer serves as a reflective surface on the buccal mucosa. The thickness of the tumor (CIS/severe dysplasia) accounts for two-thirds or more of the epithelium, starting from the basal layer.^67^ The light dosimetry shows that the dose deposit near the basal layer (1 mm) or end of tumor epithelium is $56.7\;J/{\mathrm{cm}}^{2}$. Although the Monte Carlo simulation assumed perpendicular light incidence, it was applied to the mouse model; in the oral cavity, this may not always be possible. The irradiance will be reduced by the cosine alpha of the attainable incident illumination feasible. In addition, for significantly protruding tumors, a shadow might be caused on the illumination opposing side. Hence, detailed anatomy-based simulations are required to determine the true anticipated fluorescence intensities for varying illumination and anatomical conditions. This future study also needs to perturb the tissue optical properties of the involved tissue layers and structures with their reported ranges, to complete a quantitative study of photosensitizer quantification and PDT illumination. Going forward, this information will be useful in PDT treatment planning for oral lesions by establishing a target fluence to achieve complete necrosis.

## 4 Conclusions

We have successfully shown that a handheld probe with multimodal optical imaging and a form factor designed for use in the oral cavity can be integrated with laser light delivery for image-guided PDT *in vivo*. This device combines two separate technologies into one portable, lightweight and battery-powered tool. It also maintains its previous functionality for cancer screening, allowing for autofluorescence and white-light images to be analyzed through remote diagnosis via cloud connection, as previously reported. By integrating these technologies, we seek to enable a timely image-guided intervention that front-line health workers can feasibly deliver in resource-limited settings. Going forward, we envision that the integration of imaging and PDT in the same intraoral device will enable real-time feedback for adaptive light delivery during treatment.

## Supplementary Material

10.1117/1.BIOS.2.4.042305.s01

## Data Availability

The supplementary data file contains code and supporting data. In addition, other data will be available upon reasonable request.
