# Supplementary material for: Enabling point of care optical diagnostics and treatment of oral lesions in resource-limited settings: preclinical development and evaluation of a low-cost theranostic intraoral device for image-guided photodynamic therapy
Source: Biophotonics Discov. 2025 Jul 6;2(4):042305. doi: 10.1117/1.BIOS.2.4.042305 (PMC13038290; doi:10.1117/1.BIOS.2.4.042305)
Supplement: Supplementary file 1 [file BIOS_002_042305_SD001.pdf]

Identification of colorectal malignancies enabled by phasor-based autofluorescence lifetime macroimaging and ensemble learning

João L. Lagarto,[a,*] Alberto I. Herrando,[a,b] Rafaela Rego,[c] Laura Fernández,[b] José Azevedo,[b] Hugo Domingos,[b] Pedro Vieira,[b] Amjad Parvaiz,[b] Vladislav I. Shcheslavskiy,[d,e] Pedro G. Silva,[a] Mireia Castillo-Martin,[c]

[a]Champalimaud Foundation, Biophotonics Platform, Lisbon, Portugal
[b]Champalimaud Foundation, Digestive Unit, Lisbon, Portugal
[c]Champalimaud Foundation, Pathology Service, Biophotonics Platform, Lisbon, Portugal
[d]Becker and Hickl GmbH, Berlin, Germany
[e]Privolzhsky Research Medical University, Nizhny Novgorod, Russia

Table S1. Optical configuration of the autofluorescence lifetime setup indicating spectral range of each detection channel

Detection channel	Excitation wavelength	Collection range (band-pass filters)
CH1	375 nm	380 – 420 nm
CH2		458 – 486 nm
CH3		500 – 550 nm
CH4	445 nm	458 – 486 nm
CH5		500 – 550 nm

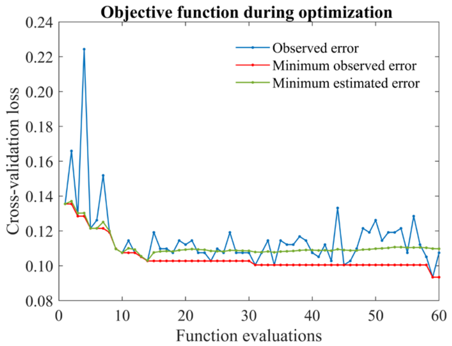

Figure S1. Observed and estimated loss during Bayesian optimization of the 5-fold cross validation model.

Table S2. Model hyperparameters using all features.

**Hyperparameters**	**Range of values**	**Value at minimum objective**
**Algorithm**	AdaBoostM1	
**Learning rate**	0 – 1	0.316
**Number of weak learners**	10 – 250	163
**Maximum of nodes per learner**	1 – 10	10

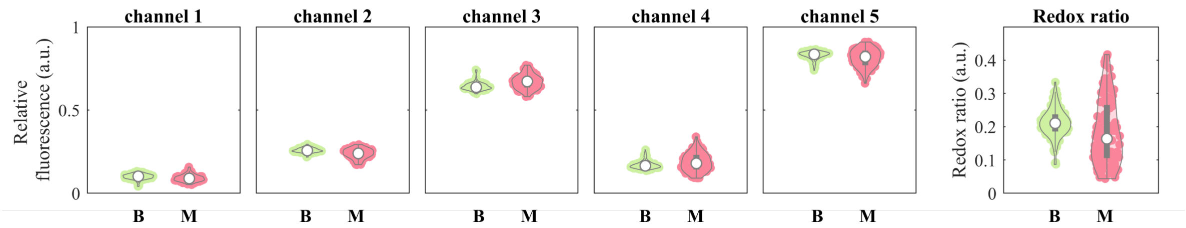

Figure S2. Relative fluorescence intensity in each detection channel and redox ratio measured in benign and malignant ROIs.

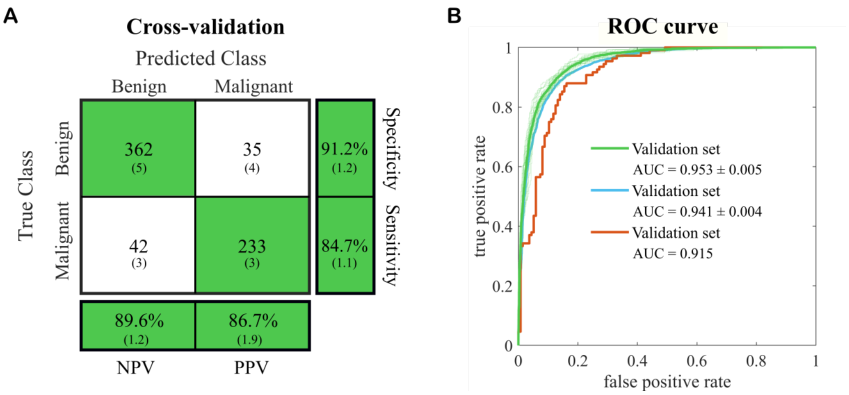

Figure S3. A) Confusion matrix obtained from training a model consisting of all samples together (training and test sets) using 5-fold cross-validation. B) Corresponding ROC curves of the combined dataset (in green). Blue and orange curves are obtained from the training and test sets, respectively, and are presented for comparison purposes. This analysis is based on cross-validation within the combined cohort and does not represent an external validation.
